# Sport als Angebot in Einrichtungen Offener Kinder- und Jugendarbeit: Studienergebnisse zur Nutzung von Sportangeboten in Jugendzentren insbesondere aus Sicht von Jugendlichen

**DOI:** 10.1007/s00103-026-04319-2

**Published:** 2026-10-20

**Authors:** Gunda Voigts

**Affiliations:** Fakultät Soziale Arbeit und Kindheitspädagogik, Hochschule für Angewandte Wissenschaften Hamburg, Alexanderstr. 1, 20099 Hamburg, Deutschland

**Keywords:** Sportsozialarbeit, Kinder- und Jugendsport, Bewegung, Jugendliche, Kinder- und Jugendhilfe, Sports social work, Children’s and youth sports, Physical activity, Adolescence, Child and youth welfare

## Abstract

Einrichtungen Offener Kinder- und Jugendarbeit sind in Deutschland nach Sozialgesetzbuch (SGB) VIII § 11 ein Ort der Kinder- und Jugendhilfe, an dem die Interessen junger Menschen im Vordergrund stehen. Jugendliche nutzen diesen Ort selbstorganisiert für sportliche Aktivitäten. Einrichtungen Offener Kinder- und Jugendarbeit bieten zugleich in ihren Angebotsprofilen Optionen zur Sportausübung für junge Menschen an.

Dieser Artikel gibt eine Übersicht über ausgewählte Befunde des Forschungsstandes zu Sport in Offener Kinder- und Jugendarbeit, zunächst mit einem Blick in die Amtliche Kinder- und Jugendhilfestatistik (2021) und die Jugendzentrumsbefragung des Deutschen Jugendinstituts (2018). Darauf aufbauend werden als Schwerpunkt dieses Beitrags Ergebnisse einer eigenen, im Jahr 2023 im Rahmen des Projektes „Move for Health“ durchgeführten qualitativen, subjektorientierten Interviewstudie und einer quantitativen Online-Befragung von Jugendlichen präsentiert. Es wird dargestellt, wie Sport in Einrichtungen Offener Kinder- und Jugendarbeit von Jugendlichen ausgeübt wird und welche Bedeutung dieser für sie einnimmt.

## Abstract (en)

According to Social Code Book VIII § 11, institutions of open child and youth work in Germany are a place of child and youth welfare where the interests of young people are in the foreground. Young people use this place in a self-organized way to practice sports.

This article provides an overview of elected findings of the state of research on sport in open child and youth work, beginning with a look at the official child and youth welfare statistics (2021) and the youth centre survey by the German Youth Institute (2018). Based on this, the focus of this article is on the results of a qualitative, subject-oriented interview study conducted in 2023 as part of the “Move for health project” and a quantitative online survey of adolescents. It is shown how sport is practiced by young people in institutions of open child and youth work and what significance it has for them.

## Einleitung

Kinder- und Jugendarbeit ist gesetzlich in Deutschland als Leistung der Kinder- und Jugendhilfe verankert. Kinder- und Jugendhilfe insgesamt zielt darauf ab, junge Menschen bis zur Vollendung des 27. Lebensjahres in ihrer Entwicklung zu unterstützen und die „Erziehung zu einer selbstbestimmten, eigenverantwortlichen und gemeinschaftsfähigen Persönlichkeit“ (Sozialgesetzbuch (SGB) VIII, § 1, 1) zu gewährleisten. Ihre individuelle und soziale Entwicklung soll gefördert, Benachteiligungen vermieden und abgebaut, Selbstbestimmung in und Teilhabe an der Gesellschaft ermöglicht und der Schutz vor Gefahren sichergestellt werden (SGB VIII, § 1, 3). Damit stellt Kinder- und Jugendarbeit als Handlungsfeld einen besonderen Ort dar. Sie soll dezidiert an „den Interessen junger Menschen anknüpfen und von ihnen mitbestimmt und mitgestaltet werden, sie zur Selbstbestimmung befähigen und zu gesellschaftlicher Mitverantwortung und zu sozialem Engagement anregen und hinführen“ (SGB VIII, § 11, 1). Sie wird so als sozialpädagogisches, institutionelles Gefüge des Aufwachsens junger Menschen in der Gesellschaft kreiert, welches explizit von ihnen selbst ausgestaltet werden soll. In Expert:innenberichten wird Kinder- und Jugendarbeit in diesem Sinne als ein Freiraum gerade in der Jugendphase verstanden, der mit Attributen wie Auszeit, Rückzugs‑, Erprobungs- oder Ermöglichungsort verbunden wird [1].

In Settings der Kinder- und Jugendarbeit treffen sich junge Menschen auch zu vielfältigen, körperlichen Aktivitäten mit Peers [2–4]. Das geschieht geplant oder spontan, punktuell oder regelmäßig, angeleitet oder selbstorganisiert, in Gleichaltrigengruppen oder altersübergreifend, in kleinen oder großen Gruppen, draußen oder drinnen. Bewegung, Spiel und Sport erscheinen nicht nur hier sowohl als „ein Interesse und eine eigene Ausdrucksform von Kindern und Jugendlichen als auch als ein Medium, um Kontakte aufzubauen, eigene Hemmschwellen abzubauen und Zugänge zu Gemeinschafts- und Bewegungserfahrungen zu schaffen“ [3].

Was mit dem Begriff Sport in der Kinder- und Jugendarbeit bzw. der Kinder- und Jugendhilfe insgesamt gemeint ist, bleibt in der Handlungspraxis dabei weitestgehend unbestimmt [7]. In einer mitverantworteten Studie wird zur Definition ein sozialkonstruktivistischer Zugang genutzt [8]. Die befragten Jugendlichen selbst bestimmen, was sie als Sport definieren. So entfernt sich das Verständnis „von einem ‚engen‘, exklusiven Sportbegriff – der idealerweise dem leistungsbezogenen, regelgeleiteten Wettkampfsport in institutionalisierten Sportarten entspricht – hin zu einem ‚weiten‘, inklusiven Sportbegriff, der vielfältige Bewegungsformen und Sinnperspektiven einschließt …“ [9].

In diesem Übersichtsartikel werden ausgewählte Befunde des Forschungsstandes zu Sport in Offener Kinder- und Jugendarbeit dargestellt. Außerdem wird auf der Grundlage von (eigenen) Forschungserkenntnissen [5, 6] nachvollzogen, wie sich Sport konkret in der Kinder- und Jugendarbeit aus der Perspektive von jungen Menschen zeigt, die Einrichtungen Offener Kinder- und Jugendarbeit nutzen.

## Offene Kinder- und Jugendarbeit

Kinder- und Jugendarbeit insgesamt ist in ihrer Organisation (Träger, Finanzierungen, Rechtskonstrukten) sowie in ihren Angebotsformen und Inhalten von einer breiten Vielfalt geprägt. Ein insbesondere von Hauptberuflichkeit, Freiwilligkeit und Offenheit gekennzeichnetes Arbeitsfeld in der Kinder- und Jugendarbeit sind Einrichtungen Offener Kinder- und Jugendarbeit. Junge Menschen besuchen diese, weil sie erstens die Einrichtungen als Ort attraktiver, kostenloser Angebote zur Freizeitgestaltung empfinden, zweitens dort Freund:innen treffen und mit ihnen Zeit verbringen können, sie drittens Mitarbeitenden begegnen, von denen sie sich anerkannt fühlen und denen sie vertrauen, und sie so viertens die Einrichtungen als einen geschützten (Rückzugs‑)Raum in ihrem Alltag erleben [10]. Sportliche Aktivitäten von jungen Menschen finden in diesen Einrichtungen vor diesem Hintergrund statt.

## Sport in Angeboten Offener Kinder- und Jugendarbeit

Eine in Deutschland zentrale, wiederkehrende empirische Erhebung zu Einrichtungen Offener Kinder- und Jugendarbeit stellt die Jugendzentrumsbefragung des Deutschen Jugendinstituts (DJI) dar. An der letzten Befragung 2018 haben sich 1350 Einrichtungen bundesweit schriftlich beteiligt [4]. Diese Einrichtungen sind in ihren Rahmungen sehr unterschiedlich, z. B. in Bezug auf Anzahl hauptberuflichen Personals, Räumlichkeiten oder Öffnungszeiten. Das gilt auch für die dortigen Angebote und Nutzungsmöglichkeiten. Einige Fragen in der Erhebung lassen Rückschlüsse auf (mögliche) Sportangebote zu. So werden Jugendzentren mit einer Liste von 31 Antwortmöglichkeiten nach den angebotenen bzw. durchgeführten Aktivitäten gefragt und diese um ein offenes Antwortfeld für eventuelle weitere Aktivitäten ergänzt. Zu den Antwortoptionen gehören die Items „sportlich-erlebnisorientierte Angebote“ und „gesundheitsorientierte Angebote“. In der Befragung 2018 haben 67 % der beteiligten Einrichtungen „sportlich-erlebnisorientierte Angebote“ angewählt (2011 waren dies noch 74 %). Die Antwortoption „gesundheitsorientiertes Angebot“ kommt 2018 erstmals vor und wird von 44 % der befragten Einrichtungen ausgewählt. 16 % der Einrichtung geben darüber hinaus an, über einen Fitnessraum zu verfügen. Das trifft auf Einrichtungen in Großstädten mit 20 % allerdings signifikant häufiger zu als in denen in Landgemeinden (7 %) oder kleineren Kleinstädten (11 %). Einen Außenbereich – also z. B. Grünflächen oder Plätze, die Ballsport oder Rückschlagspiele ermöglichen – haben demnach 83 % aller beteiligten Einrichtungen [4].

Weitere regelmäßige Daten zur Kinder- und Jugendarbeit werden in der Amtlichen Kinder- und Jugendhilfestatistik bei freien und öffentlichen Trägern bundesweit durch die Arbeitsstelle Kinder- und Jugendhilfestatistik im Forschungsverbund DJI/TU Dortmund erhoben. In dieser Befragung zielt eine Frage im Kontext einrichtungsbezogener, offener Kinder- und Jugendarbeit auf Themenschwerpunkte ab, die neben dem offenen Angebotsbereich verfolgt werden. Die Antwortoptionen „Spiel“ (47,6 %) und „Sport“ (27,3 %) erhalten dabei in der Erhebung 2021 die beiden höchsten Zustimmungswerte [2].

Aus diesen beiden Erhebungen kann resümiert werden, dass sportliche Aktivitäten im Angebotsprofil von Einrichtungen Offener Kinder- und Jugendarbeit einen empirisch relevanten Stellenwert einnehmen. Insgesamt werden jedoch Forschungsergebnisse zum Sport in der Offenen Kinder- und Jugendarbeit als wenig ausgeprägt bewertet [11–13]. Dies scheint damit zusammenzuhängen, dass in der gesamten Kinder- und Jugendhilfe „die pädagogische Bedeutung von Sport … oft nicht erkannt“ [14] wird.

Von dieser Analyse ausgehend wurde im wissenschaftlichen Modul „Potenziale von Bewegung, Spiel und Sport für ein gesundes Aufwachsen in Deutschland“ [5] des Projektes „Move for Health“ der Deutschen Sportjugend ein qualitatives Teilforschungsprojekt mit dem Titel „Soziale Teilhabe durch Sportangebote in Einrichtungen der Offenen Kinder- und Jugendarbeit“ [6] initiiert. Parallel wurden in der im Gesamtprojekt durchgeführten bundesweiten, repräsentativen Befragung von Jugendlichen Frageitems mit Bezug zu Sport in der Kinder- und Jugendarbeit aufgenommen [5]. Die Erkenntnisse dieser beiden Erhebungen werden im Folgenden referiert. Sie legen die Perspektiven junger Menschen selbst auf Sport in der Kinder- und Jugendarbeit dar und füllen damit eine bisher vorhandene Forschungslücke.

## Ergebnisse der Online-Befragung von Jugendlichen zur Bedeutung Offener Kinder- und Jugendarbeit für sportliche Aktivitäten

Im Zentrum der Gesamtstudie „Potenziale von Bewegung, Spiel und Sport für ein gesundes Aufwachsen in Deutschland“ [5] im Projekt „Move for Health“ stand die psychische Gesundheit von Kindern und Jugendlichen und deren Zusammenhang mit der Ausübung von Sportaktivitäten. 2023 wurden in einer bundesweiten, für Deutschland repräsentativen Online-Befragung 1978 auswertbare Datensätze von Jugendlichen zwischen 13 bis 17 Jahren und deren Eltern erzeugt und analysiert [5]. Einrichtungen Offener Kinder- und Jugendarbeit als Ort, an dem Kinder und Jugendliche Sport ausüben, waren dabei punktuell in Fragen aufgenommen worden.

Von den beteiligten Jugendlichen zwischen 13 und 17 Jahren geben 9 % an, dass sie Sportangebote in Settings Offener Kinder- und Jugendarbeit nutzen. 2 % der Jugendlichen und 3 % der Kinder benennen, dass sie dort die aus ihrer Sicht wichtigste Sport- bzw. Bewegungsaktivität ausüben. Mit den Sportangeboten in der Offenen Kinder- und Jugendarbeit werden laut der Studie eher junge Menschen erreicht, die seltener in Sportvereinen organisierten Sport betreiben, beispielsweise körperlich beeinträchtigte Jugendliche oder Schüler:innen, die Haupt- oder Mittelschulen besuchen [15].

Die „Lust auf das Sportangebot“ sowie „Freund:innen zu treffen“, die auch zu den Angeboten kommen, zeigen sich als zentrale Motive, warum die Befragten Sport in Einrichtungen der Offenen Kinder- und Jugendarbeit nachgehen. Hier besteht kein grundlegender Unterschied zu Motivlagen der Nutzung von Angeboten in Sportvereinen. Allerdings wird mit Blick auf die Sportausübung in Einrichtungen Offener Kinder- und Jugendarbeit im Vergleich zu Sportvereinen häufiger geäußert, dass dort mitentschieden werden kann, was gemacht wird, und dass „etwas ausprobiert“ werden kann: 15 % der Jungen und 14 % der Mädchen benennen, dass sie an Sportangeboten in der Offenen Kinder- und Jugendarbeit teilnehmen, weil sie dort „mitentscheiden“ können. Bezüglich einer Mitgliedschaft im Sportverein ist das nur bei 5 % der Jungen und 10 % der Mädchen ein Motiv. 25 % der Jungen und 19 % der Mädchen teilen mit, dass sie an Sportangeboten in der Offenen Kinder- und Jugendarbeit partizipieren, weil sie dort „etwas ausprobieren“ können, bei Sportvereinen geben das 9 % der Jungen und 17 % der Mädchen an [6, 15].

Sportvereine scheinen davon zu profitieren, dass sie deutlich besser zu erreichen sind. 60 % der Jungen und 52 % der Mädchen geben an, dass sie Mitglied im Verein seien, weil das Sportangebot „gut erreichbar“ sei. Mit Blick auf Sportangebote in Einrichtungen Offener Kinder- und Jugendarbeit geben das nur 22 % der Jungen und 15 % der Mädchen an. Ein deutlicher Unterschied in den Antworten besteht auch bei der Frage, ob Eltern zu einer Teilnahme motiviert haben. Für Sportvereine geben das 31 % der Jungen und 41 % der Mädchen an, bei der Offenen Kinder- und Jugendarbeit hingegen nur 13 % der Jungen und 8 % der Mädchen [6, 15].

Als Resümee der quantitativen Befragung innerhalb der Studie „Move for Health“ lässt sich festhalten, dass Einrichtungen Offener Kinder- und Jugendarbeit für einen Teil der 13- bis 17-jährigen Jugendlichen ein bedeutender Sportort sind. Zugleich sind mit 42,5 % knapp 5‑mal so viele junge Menschen in einem Sportverein aktiv [15]. Eine Erkenntnis ist, dass Offene Kinder- und Jugendarbeit mittunter eher Zielgruppen ansprechen und für Sportaktivitäten gewinnen kann, die von Sportvereinsangeboten weniger gut erreicht werden.

## Bedeutung von Sport in Offener Kinder- und Jugendarbeit aus Sicht von Jugendlichen

Im Rahmen der qualitativen Teilstudie „Soziale Teilhabe durch Sportangebote in Einrichtungen der Offenen Kinder- und Jugendarbeit“ des Projektes „Move for Health“ wurden im Sommer 2023 Jugendliche, die Einrichtungen Offener Kinder- und Jugendarbeit in Hamburg nutzen, in Interviews befragt.^1^ Ziel war es, neues Wissen zu Sport in diesem Handlungsfeld zu generieren. Von Interesse erschienen Angebotsprofile der Einrichtungen, Vernetzungen im Sozialraum vor allem auch mit Sportvereinen, Zugänge und Zugangsbarrieren zu Angeboten sowie die Frage, ob Sport in Einrichtungen Offener Kinder- und Jugendarbeit für Jugendliche als ein stabilisierender Faktor im Aufwachsen eingeordnet werden kann. Dabei wurden besonders junge Menschen aus sozial benachteiligten Lebenslagen in den Fokus genommen.

Mit einem Interviewleitfaden, der 23 Fragen in 5 Themenblöcken enthielt, wurden in 9 unterschiedlichen Einrichtungen in Hamburg Gespräche mit 25 Jugendlichen im Alter von 14 bis 25 Jahren (Schwerpunkt 14 bis 19 Jahre) geführt. Alle Jugendlichen haben zum Befragungszeitpunkt in den Einrichtungen Sport ausgeübt. Ausgewertet wurde das transkribierte Interviewmaterial mit der Methodik der qualitativen Inhaltsanalyse nach Kuckartz [17].

Zur genaueren Beschreibung des Samples wurden die Jugendlichen mit einem gesonderten Fragebogen um persönliche Angaben gebeten. Daraus lässt sich ableiten, dass 18 der Befragten sich als männlich definieren, 7 als weiblich. Ein Drittel der Jugendlichen gab an, nicht in Deutschland geboren zu sein. Bei mehr als der Hälfte der Befragten sind weder Mutter noch Vater in Deutschland geboren. Fast alle Jugendlichen leben nach den eigenen Angaben in Mietwohnungen und ihre Eltern verfügen über eher niedrige Bildungsabschlüsse.

### Nutzung von Sportangeboten: Motive, Sportarten und Häufigkeit

In den Interviews verbinden die befragten jungen Menschen „Sport“ zuallererst mit positiven Attributen und Gefühlen. Beschreibungen sind „Spaß mit Freund:innen“, „Gemeinschaft“, „Durchhaltevermögen“, „Teamarbeit“ oder „Zusammenhalt“. „Stärke“, „Kraft“, Optionen der „Entwicklung“, „Begeisterung“ und „Stolz“ werden ebenfalls angeführt. „Gesundheitlich fit“ zu werden oder zu bleiben, sich „selbst weiterzuentwickeln“ oder „gute Zeit mit Freunden“ zu verbringen, werden als wichtige Motive genannt, selbst Sport zu treiben.

Ein großer Teil der Jugendlichen übt in den Einrichtungen Offener Kinder- und Jugendarbeit nicht nur eine Sportart aus, sondern geht dort unterschiedlichen Aktivitäten nach. Besonders spontan zugängliche Optionen wie Basketballkörbe, Kicker, Billard oder Tischtennisplatten spielen dabei eine Rolle. Als hauptsächlich ausgeübte Sportarten werden Basket‑, Fuß- und Volleyball sowie Kampfsportarten und Fitness/Kraftsport, Tanzen und Kickern benannt.

Die Bewegungsaktivitäten werden sowohl in festen oder offenen Gruppen, zu festen Zeiten oder spontan, selbstorganisiert oder als Angebot von Trainer:innen durchgeführt. Etwas mehr als die Hälfte der Jugendlichen ist in Angeboten aktiv, die von Erwachsenen betreut oder gestaltet werden (Trainer:innen, Betreuer:innen, Honorar- und Fachkräfte). Prinzipiell werden alle Angebote als offen bezeichnet, auch wenn vereinzelt Anmeldungen gerne gesehen seien. Einige Jugendliche übernehmen selbst Verantwortung für Angebote, bereiten diese vor und leiten andere, manchmal jüngere Teilnehmende an. Sie sprechen davon, darin Anerkennung zu erfahren und es gut zu finden, anderen etwas beibringen zu können und von diesen akzeptiert, manchmal auch bewundert zu werden.

Die Häufigkeit der Nutzung der Angebote bzw. der Einrichtung insgesamt ist sehr unterschiedlich. Einige Befragte kommen ausschließlich für die Sportangebote zu festen Zeiten, andere nutzen die Einrichtung Offener Kinder- und Jugendarbeit auch für andere Aktivitäten, wie z. B. „mit Freund:innen abhängen“ oder für eine Beratung von den Fachkräften. Die meisten Angebote finden in oder rund um die Einrichtung statt, nur sehr selten wird von Kooperationen mit Sportvereinen berichtet.

Erzählt wird, dass das Material für den Sport in der Regel kostenfrei von der Einrichtung zur Verfügung gestellt werde und dass das wichtig sei. Das gilt nicht nur für benötigtes Sportmaterial wie „Bälle“, „Schläger“ oder „Gewichte“, sondern meistens auch für „Trikots“ oder andere benötigte (persönliche) Ausrüstung. Gesprochen wird von den Befragten allerdings darüber, dass Material manchmal veraltet oder kaputt sei und kein Geld für Neuanschaffungen in der Einrichtung vorhanden sei. Es wird der Wunsch geäußert, dass sich das ändere.

### Zugang und Atmosphäre: Soziale Kontakte als entscheidende Basis der Nutzung

In den Interviews zeigt sich, dass Sportangebote zum einen häufig ein erster Zugang in die Einrichtungen Offener Kinder- und Jugendarbeit sind. Sport dient als „Türöffner“. Zum anderen gibt es Jugendliche, die durch ihren Besuch in den Einrichtungen mit Sportangeboten in Kontakt gekommen sind. Für sie war der Besuch, meistens initiiert durch Freund:innen, wiederum der „Türöffner“ zum Sporttreiben. Freie Sportangebote ohne Verpflichtung nehmen dabei eine besondere Rolle ein. Darüber hinaus benennt etwa ein Viertel der Befragten Schulkooperationen, die zur Nutzung der Einrichtung und der Sportangebote dort geführt haben.

Als Unterschied zum Sportreiben in Sportvereinen wird von den Jugendlichen herausgestellt, dass dort mehr sportlich ambitionierte Ziele verfolgt werden müssten und die Anforderungen und die einzugehenden Verpflichtungen, auch in Form von Verträgen, größer seien. Ein großer Teil der Jugendlichen ist oder war allerdings auch in einem Sportverein aktiv.

Die Beziehungen zu anderen Jugendlichen, zu den Trainer:innen oder Mitarbeitenden in den Einrichtungen Offener Kinder- und Jugendarbeit sind ein zentrales Merkmal der Ausübung von Sport dort. Das Vertrauen zueinander, der hohe Wohlfühlfaktor und der gegenseitige Respekt werden als besonders herausgestellt und bieten laut den Jugendlichen einen geschützten und ermunternden Rahmen zu körperlichen Aktivitäten. Der Sport erzeuge auf dieser Basis Gemeinschaft und biete Raum, voneinander und miteinander zu lernen. Trainer:innen und Mitarbeitende werden über die Sportangebote hinaus als Ansprechpersonen geschätzt, scheinen Mitbestimmungsoptionen zu bieten und dienen bei Bedarf als Ratgebende und Unterstützer:innen bei anderen (persönlichen) Angelegenheiten.

Die Jugendlichen berichten davon, sich in den Einrichtungen „sicher“ zu fühlen und sich daher „ausprobieren“ zu können. Die Freiwilligkeit der Teilnahme und die Mitbestimmungsoptionen – nicht nur bei den Sportangeboten – werden in den Interviews immer wieder als große Besonderheit hervorgehoben. Ebenso die Offenheit für die Vielfalt der jungen Menschen in der Einrichtung, die mit dem Gefühl des „Willkommen-Seins“ verbunden wird.

Sportangebote in Einrichtungen Offener Kinder- und Jugendarbeit leisten so einen Beitrag als stabilisierender Faktor in der Alltagsbewältigung. Einrichtungsbesuche an sich können somit eine besondere Bedeutung bei der Bewältigung individueller Krisen haben.

## Fazit

Sowohl die qualitative Befragung jugendlicher Nutzer:innen von Offener Kinder- und Jugendarbeit in Hamburg als auch die Ergebnisse der bundesweiten Online-Befragung legen dar, dass Einrichtungen Offener Kinder- und Jugendarbeit Relevanz bei der Ausübung von (gemeinschaftlichem) Sport im Jugendalter haben. Die Einrichtungen sprechen mit ihren niedrigschwelligen, nicht an Mitgliedschaft gebundenen, kostenfreien Sportangeboten Jugendliche an, die Sportvereine manchmal weniger gut erreichen können. Sie fördern so sportliche Aktivität im Jugendalter. Die Möglichkeiten der spontanen und freiwilligen Nutzung ohne (Leistungs‑)Druck sind dabei von Bedeutung und stellen ein wesentliches Merkmal dar, ebenso die sozialen Kontakte, die erlebte Anerkennung und Mitbestimmung.

Anhand der zusammengetragenen Erkenntnisse lässt sich festhalten, dass sowohl Sportangebote als auch das spontane Entstehen von Sportaktivitäten zum Nutzungsspektrum von Einrichtungen Offener Kinder- und Jugendarbeit gehören. Gleichermaßen verstehen die befragten Jugendlichen diese Einrichtungen als Orte, an denen sie gerne Sport ausüben und Angebote mitgestalten. Einrichtungen Offener Kinder- und Jugendarbeit sollte vor diesem Hintergrund mehr Beachtung in den Diskussionen über die Bedeutung von Sport und Bewegungsförderung im Jugendalter gegeben werden.

## Data Availability

Für diesen Artikel wurden keine Datensätze erstellt oder analysiert.

## Footnotes

1 Die im Folgenden referierten Erkenntnisse sind umfassender in 2 Veröffentlichungen [6, 16] der Autorin und einer weiteren Forscherin nachzulesen. Zur besseren Lesbarkeit wird darauf verzichtet, wiederholend auf diese Originalquellen bei Nennung der Ergebnisse zu verweisen.
